# Anionic block copolymer vesicles act as Trojan horses to enable efficient occlusion of guest species into host calcite crystals†
†Electronic supplementary information (ESI) available: Experimental details and characterization methods, *e.g.* GPC, DLS, SAXS data, optical microscopy, fluorescence microscopy, SEM images, Raman studies and TGA analysis. See DOI: 10.1039/c8sc03623c


**DOI:** 10.1039/c8sc03623c

**Published:** 2018-09-10

**Authors:** Yin Ning, Daniel J. Whitaker, Charlotte J. Mable, Matthew J. Derry, Nicholas J. W. Penfold, Alexander N. Kulak, David C. Green, Fiona C. Meldrum, Steven P. Armes

**Affiliations:** a Department of Chemistry , University of Sheffield , Brook Hill, South Yorkshire S3 7HF , Sheffield , UK . Email: Y.Ning@sheffield.ac.uk ; Email: s.p.armes@sheffield.ac.uk; b School of Chemistry , University of Leeds , Woodhouse Lane , Leeds , LS2 9JT , UK

## Abstract

We report a versatile ‘Trojan Horse’ strategy using highly anionic poly(methacrylic acid)–poly(benzyl methacrylate) vesicles to incorporate two types of model payloads, *i.e.* either silica nanoparticles or an organic dye (fluorescein), within CaCO_3_ (calcite). Uniform occlusion of silica-loaded vesicles was confirmed by scanning electron microscopy, while thermogravimetry studies indicated extents of vesicle occlusion of up to 9.4% by mass (∼33% by volume). Efficient dye-loaded vesicle occlusion produces highly fluorescent calcite crystals as judged by fluorescence microscopy. In control experiments, silica nanoparticles alone are barely occluded, while only very weakly fluorescent calcite crystals are obtained when using just the fluorescein dye. This new ‘Trojan Horse’ strategy opens up a generic route for the efficient occlusion of various nanoparticles and organic molecules within inorganic host crystals.

## Abstract 2

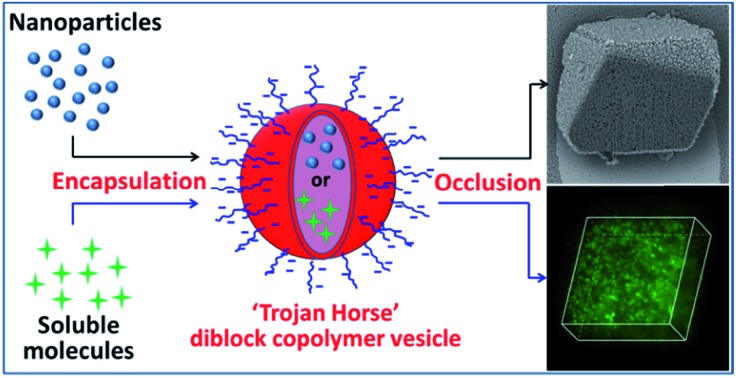
‘Trojan Horse’ anionic poly(methacrylic acid)–poly(benzyl methacrylate) vesicles enable efficient incorporation of either nanoparticles or soluble small molecules within calcite.

## Introduction

Nanoparticle occlusion within host crystals such as calcite,^1^–^5^ ZnO^6^–^9^ or Cu_2_O^10^,^11^ is an emerging research topic of considerable growing interest. It offers a promising route to new classes of nanocomposite materials with enhanced mechanical or other physical properties,^12^,^13^ as well as providing a useful model system to aid our understanding of biomineralization.^14^–^17^ However, only nanoparticles with appropriate surface chemistry,^18^–^21^ or when subjected to physical confinement,^10^,^11^ can be incorporated within inorganic host crystals. Such constraints currently prevent broader application of this approach for the development of new classes of nanocomposite materials. Thus a universal strategy that enables the efficient occlusion of nanoparticles (and preferably also organic molecules) within inorganic crystals would be highly desirable.

Over the past decade or so, polymerization-induced self-assembly (PISA) has been demonstrated to be a powerful route for the synthesis of diblock copolymer nano-objects with various morphologies (*e.g.* spheres, worms or vesicles).^22^–^37^ PISA enables nano-objects to be prepared at high solids, which offers a decisive advantage over traditional post-polymerization processing techniques that are typically conducted at high dilution (<1% copolymer).^38^,^39^ It is well-known that targeting longer core-forming blocks usually favors vesicle formation during PISA.^40^ Recently, both silica and globular proteins such as BSA or enzymes have been encapsulated *in situ* within non-ionic poly(ethylene glycol)–poly(2-hydroxypropyl methacrylate) (PEG–PHPMA)^41^–^47^ or poly(glycerol monomethacrylate)–poly(2-hydroxypropyl methacrylate) (PGMA–PHPMA)^48^,^49^ vesicles prepared *via* PISA in aqueous media. These prior studies inspired us to ask an important question: can we “smuggle” nanoparticles or (macro)molecules into host inorganic crystals by using similar vesicles as a delivery vehicle?

**Scheme 1:**
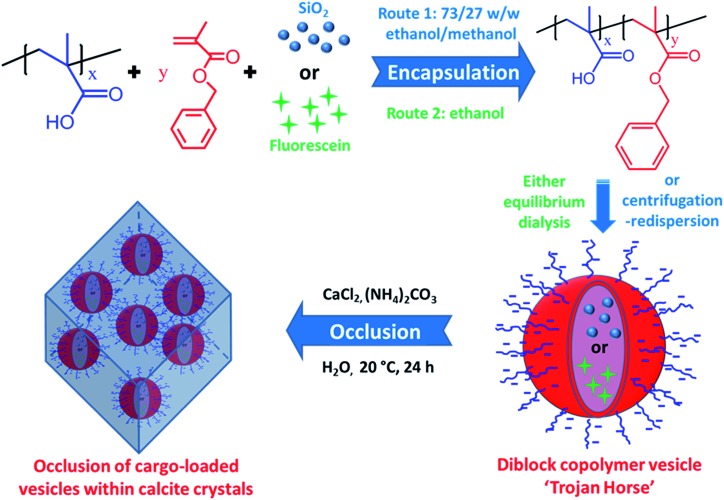
Synthesis of poly(methacrylic acid)_*x*_–poly(benzyl methacrylate)_*y*_ (PMAA_*x*_–PBzMA_*y*_) vesicles at 20% w/w solids in the presence of either silica nanoparticles (Route 1) or fluorescein (Route 2) *via* RAFT dispersion polymerization of BzMA at 70 °C for 24 h. Excess silica nanoparticles or fluorescein dye were removed *via* either centrifugation–redispersion cycles (for the silica nanoparticles) or equilibrium dialysis (for the fluorescein dye). Schematic cartoon showing the occlusion of either silica-loaded or fluorescein-loaded PMAA_*x*_–PBzMA_*y*_ vesicles within calcite (CaCO_3_) crystals.

Herein we exploit diblock copolymer vesicles as ‘Trojan Horses’ to occlude inorganic nanoparticles or organic molecules into host inorganic crystals. More specifically, either silica nanoparticles or fluorescein were first encapsulated within poly(methacrylic acid)–poly(benzyl methacrylate) (PMAA–PBzMA) diblock copolymer vesicles *via* reversible addition-fragmentation chain transfer (RAFT) alcoholic dispersion polymerization (see Scheme 1). Subsequently, the silica- or dye-loaded vesicles were transferred into aqueous solution and thereby acquired substantial anionic surface charge *via* ionization of the carboxylic acid groups on the PMAA stabilizer chains. Synthesis of calcite (CaCO_3_) single crystals in the presence of such vesicles in weakly alkaline solution (pH ∼ 9) leads to occlusion of the anionic vesicles. In principle, this new ‘Trojan Horse’ approach provides a generic and versatile route to occlude a wide range of nanoparticles, macromolecules or small molecules within host crystals.

## Results and discussion

Unsurprisingly, no occlusion was observed for the crystals precipitated in the presence of the non-ionic PEG–PHPMA and PGMA–PHPMA vesicles (see Fig. S1 and S2, ESI†). We therefore synthesized anionic vesicles, where these were expected to bind strongly to the growing calcite crystals.^14^ However, direct PISA synthesis of such vesicles in aqueous solution is rather challenging owing to strong mutual repulsion between the polyelectrolytic stabilizer chains.^18^,^50^ In principle, this problem can be addressed by using a binary mixture comprising an anionic and non-ionic stabilizer block.^51^ However, this approach necessarily reduces the anionic surface charge density, which may prevent or reduce efficient occlusion. Fortunately, appropriate vesicles can be prepared *via* RAFT alcoholic dispersion polymerization,^52^ because stabilizer blocks such as PMAA remain essentially non-ionized in such solvents.^53^,^54^

**Fig. 1:**
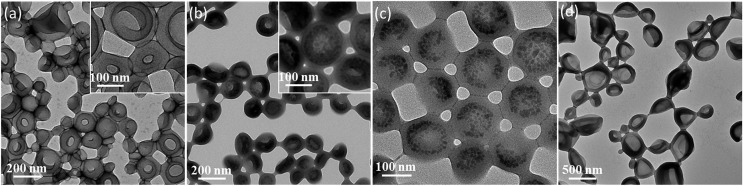
Transmission electron microscopy (TEM) images recorded for: (a) stained PMAA_69_–PBzMA_200_ vesicles prepared in the absence of any silica nanoparticles; (b) stained PMAA_69_–PBzMA_200_ vesicles prepared in the presence of silica nanoparticles (after removing excess silica by multiple centrifugation–redispersion cycles); (c) unstained PMAA_69_–PBzMA_200_ vesicles, clearly showing the encapsulation of silica within the vesicle lumen; (d) stained PMAA_62_–PBzMA_300_ vesicles prepared in the presence of fluorescein dye (after removing excess dye *via* equilibrium dialysis). The vesicle aggregation observed in these TEM images is just a drying artefact.

Two types of vesicles were prepared in the present study (see Scheme 1). In Route 1, a PMAA_69_ macro-CTA was synthesized in ethanol using a trithiocarbonate-based RAFT agent and then chain-extended with benzyl methacrylate (BzMA) in the presence of silica nanoparticles in a 73/27 w/w ethanol/methanol mixture. In Route 2, a PMAA_62_ macro-CTA was prepared using a dithiobenzoate-based RAFT agent in ethanol prior to chain-extension with BzMA in the presence of a model dye (fluorescein) (see Fig. S3†). In each case, more than 99% BzMA conversion was obtained after 24 h at 70 °C by ^1^H NMR spectroscopy studies. The silica nanoparticles (or fluorescein) are encapsulated within the vesicle lumen during the PISA synthesis. Excess silica nanoparticles were removed by centrifugation (with the sedimented vesicles being redispersed in deionized water), while free dye was removed by equilibrium dialysis against deionized water. Gel permeation chromatography (GPC) analysis indicated that both PMAA and PMAA–PBzMA had relatively narrow molecular weight distributions, confirming that the presence of silica nanoparticles (or dye) did not adversely affect the PISA synthesis (Fig. S4†). [N.B. These PMAA–PBzMA diblock copolymers were exhaustively methylated before GPC analyses to avoid unwanted interaction between the anionic PMAA block and the GPC column.] Well-defined PMAA–PBzMA vesicles were obtained regardless of the presence of either additive (see Fig. 1 and S5†). Interestingly, fluorescein-loaded PMAA_62_–PBzMA_300_ vesicles (647 ± 193 nm diameter) were significantly larger than silica-loaded PMAA_69_–PBzMA_200_ vesicles (232 ± 60 nm diameter), as determined by dynamic light scattering, see Table S1 and Fig. S6.† This may be related to the use of methanol co-solvent for the latter system, which is a better solvent for the PMAA stabilizer chains than ethanol. The commercial methanolic silica sol used in this study exhibited a mean diameter of approximately 12 nm, as determined by TEM (see Fig. S5a†). The electron contrast between the PMAA_69_–PBzMA_200_ vesicles and the silica nanoparticles is sufficiently high for TEM to provide direct evidence of silica encapsulation (compare Fig. 1a and b). The TEM image obtained for the unstained vesicles provided convincing evidence that silica nanoparticles were indeed encapsulated within the lumen (see Fig. 1c and S7†). Successful silica encapsulation was also confirmed by small angle X-ray scattering (SAXS), which revealed a structure factor at *q* ∼ 0.13 nm^–1^ (see Fig. S7c†).^48^ Thermogravimetric analysis (TGA) indicated that the silica-loaded vesicles contained 11% silica by mass (see Fig. S8†), which suggests an encapsulation efficiency of approximately 25% for these nanoparticles.

Calcite single crystals were prepared at pH ∼ 9 by the ammonia diffusion method at 20 °C for 24 h.^55^,^56^ Well-defined rhombohedral calcite crystals were obtained in the absence of vesicles (see Fig. S9a and b†). In contrast, calcite crystals prepared in the presence of silica-loaded PMAA_69_–PBzMA_200_ vesicles were uniformly decorated with adsorbed (or partially occluded) vesicles (Fig. S9c and d†). Optical microscopy confirmed that these crystals were opaque, unlike the transparent control crystals prepared in the absence of any additives (Fig. S10†). This is ascribed to light scattering arising from the refractive index difference between the vesicles (RI ∼ 1.56 PBzMA) and the calcite (RI ∼ 1.486). Raman spectroscopy was used to confirm the crystal polymorph: bands at 1088 (*v*_1_), 713 (*v*_4_), 281 and 155 (lattice modes) cm^–1^ are well-known to be characteristic of calcite (see Fig. S11†).^57^ Interestingly, two new Raman bands at 1004 and 1032 cm^–1^ were observed for both the empty vesicle/calcite and silica-loaded vesicle/calcite nanocomposites. These signals are assigned to the symmetric breathing vibration mode and in-plane C–H bending of the aromatic rings in the core-forming PBzMA block.^58^

**Fig. 2:**
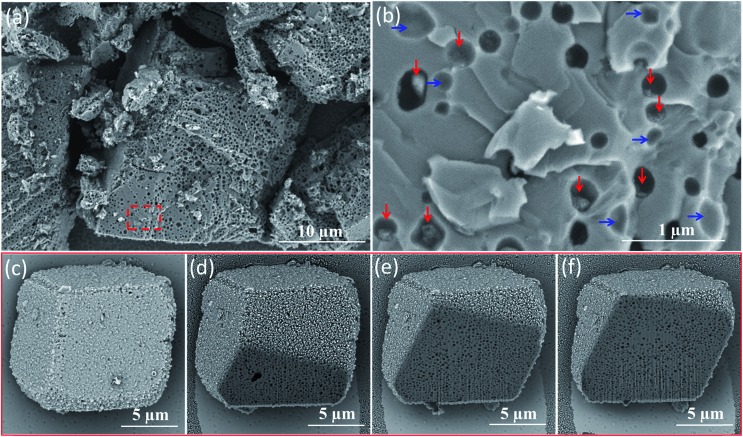
(a) SEM image obtained for a fractured calcite crystal occluded with silica-loaded PMAA_69_–PBzMA_200_ vesicles. (b) Higher magnification SEM image of the area indicated by the red rectangle shown in (a). The red arrows indicate aggregated silica nanoparticles while the blue arrows indicate empty cavities. (c–f) Four SEM images for the same vesicle/calcite nanocomposite showing a sequence of cross-sections recorded over time during the continuous focused ion beam (FIB) etching of an individual calcite crystal. This series of images indicates that uniform, dense vesicle occlusion has been achieved throughout this host matrix.

Direct evidence for uniform vesicle occlusion within calcite was obtained by imaging the fractured crystals. Densely-packed, well-separated voids are observed, see Fig. 2a and b. These SEM images were recorded without any sputtered metal coating. Poor electron contrast renders the PMAA_69_–PBzMA_200_ vesicles invisible under such conditions. However, dried aggregates of silica nanoparticles can be visualized within some of the cavities, see red arrows in Fig. 2b. Empty cavities were also observed, as indicated by blue arrows. This is because only some of the silica-loaded vesicles remain in each half of the fractured crystal surface. The dimensions of both types of cavities are consistent with the original vesicle dimensions as determined by TEM and DLS. Moreover, all cavities are isolated from their neighbors, suggesting that the vesicles remained colloidally stable during their occlusion. This observation also confirmed that the apparent vesicle aggregation suggested in Fig. 1 is simply a drying artefact. Indeed, well-dispersed silica-loaded vesicles could be observed by simply diluting these dispersions using a 6 mM CaCl_2_ aqueous solution for the TEM grid preparation (see Fig. S12†). The uniform distribution of these vesicles throughout the host crystal was confirmed by examining a sequence of images obtained for a vesicle-loaded crystal during its focused ion beam (FIB) milling, as shown in Fig. 2c–f.

TGA analyses indicated that the extent of vesicle occlusion is 9.4% by mass, which corresponds to 32.6% by volume (see Fig. S8†). In a control experiment conducted in the presence of silica nanoparticles alone (*i.e.*, in the absence of vesicles), SEM analysis of fractured calcite crystals suggests that only trace levels of silica occlusion can be achieved. Moreover, TGA analyses of such calcite crystals are essentially indistinguishable from those of pure calcite, which again indicates negligible silica occlusion (see Fig. S13†).

**Fig. 3:**
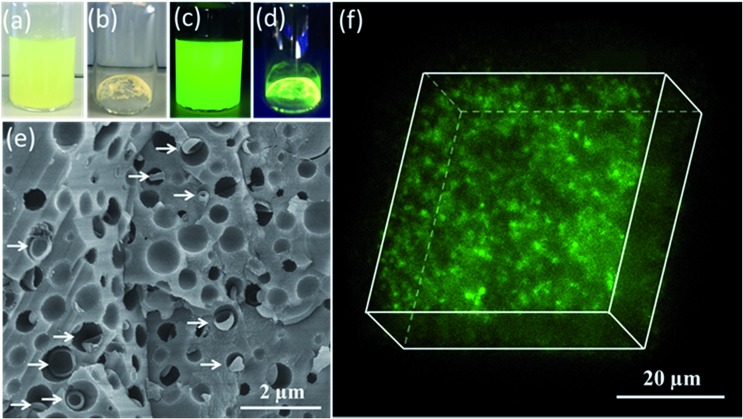
Digital micrographs obtained for (a) an aqueous dispersion of fluorescein-loaded vesicles and (b) fluorescein-loaded vesicles/calcite nanocomposites; (c) and (d) are the corresponding images recorded during UV irradiation (365 nm, 6 W lamp). (e) SEM image recorded for a fractured calcite crystal containing the uniformly-occluded fluorescein-loaded PMAA_62_–PBzMA_300_ vesicles. (N.B. This latter sample was sputter-coated with gold, enabling the vesicles to be visualized, see white arrows.) (f) Confocal fluorescence image recorded for fluorescein-loaded PMAA_62_–PBzMA_300_ vesicles occluded within a calcite crystal. This z-stacked image provides good evidence for uniform occlusion.

Having demonstrated the efficient occlusion of silica-loaded vesicles within calcite, we then extended our ‘Trojan Horse’ strategy to include soluble organic molecules because incorporating such additives into host crystals is of considerable interest.^59^–^77^ More specifically, fluorescein was employed as a model small molecule payload. In this case, a longer membrane-forming block was targeted to achieve relatively thick membranes and hence retard dye diffusion from the vesicle lumen.^78^ As shown in Fig. 3c, the fluorescein-loaded vesicles remained strongly fluorescent after removal of the excess free dye *via* dialysis. Occlusion of such dye-loaded vesicles produces highly fluorescent calcite crystals (see Fig. 3d). Moreover, a high density of occluded vesicles was observed throughout the host matrix when imaging a fractured calcite crystal (see Fig. 3e) while the attempted incorporation of fluorescein alone leads to minimal occlusion (see Fig. S14†). Given the relatively large size of the fluorescein-loaded PMAA_62_–PBzMA_300_ vesicles (∼647 nm diameter as judged by DLS), confocal fluorescence microscopy enabled individual fluorescein-loaded PMAA_62_–PBzMA_300_ vesicles to be detected, and confirmed their uniform distribution throughout the calcite crystal (see Fig. 3f).

## Conclusions

In summary, we demonstrate that either inorganic nanoparticles or organic dyes can be loaded within PMAA–PBzMA vesicles during their PISA synthesis *via* RAFT dispersion polymerization. The anionic PMAA stabilizer chains ensure that the silica- or dye-loaded vesicles can be densely and uniformly occluded throughout calcite single crystals. Thermogravimetry studies indicate extents of occlusion of up to 32.6% by volume can be achieved for the silica-loaded vesicles, and incorporating fluorescein-loaded vesicles into calcite leads to highly fluorescent host crystals. In principle, this new ‘Trojan Horse’ strategy provides an efficient and generic route to prepare a wide range of novel nanocomposite materials.

## Conflicts of interest

There are no conflicts to declare.

## Supplementary Material

Supplementary information
