# Supplementary material for: Anionic block copolymer vesicles act as Trojan horses to enable efficient occlusion of guest species into host calcite crystals
Source: Chem Sci. 2018 Sep 10;9(44):8396–401. doi: 10.1039/c8sc03623c (PMC6243646; doi:10.1039/c8sc03623c)
Supplement: Supplementary file 1 [file SC-009-C8SC03623C-s001.pdf]

Electronic Supplementary Material (ESI) for Chemical Science.
This journal is © The Royal Society of Chemistry 2018

Supporting Information

Anionic block copolymer vesicles act as Trojan horses to enable efficient occlusion of guest species into host calcite crystals

Yin Ning,*,† Daniel J. Whitaker,† Charlotte J. Mable,† Matthew J. Derry,† Nicholas J. W. Penfold,† Alexander N. Kulak,‡ David C. Green,‡ Fiona C. Meldrum,‡ and Steven P. Armes*,†

† Department of Chemistry, University of Sheffield, Brook Hill, Sheffield, South Yorkshire S3 7HF, UK.

‡ School of Chemistry, University of Leeds, Woodhouse Lane, Leeds, LS2 9JT, UK.

1. Experimental Section

1.1. Materials

Methacrylic acid (MAA), benzyl methacrylate (BzMA), 4,4'-azobis(4-cyanovaleric acid) (ACVA; 99%) and 2,2'-azobis[2-(2-imidazolin-2-yl)propane]dihydrochloride (VA-044) were purchased from Wako Pure Chemical Industries, Ltd (Osaka, Japan). 4-Cyano-4-(phenylcarbonothioylthio)pentanoic acid (CPCP), 2-cyano-2-propyl dithiobenzoate (CPDB), fluorescein, ammonium carbonate and calcium chloride hexahydrate were all purchased from Sigma-Aldrich (UK) and used as received. Glycerol monomethacrylate (GMA; 99.8%) was supplied by GEO Specialty Chemicals (Hythe, UK) and used without further purification. 4-Cyano-4-(2-phenylethane sulfanylthiocarbonyl)sulfanylpentanoic acid (PETTC) was prepared according to the protocol described by Semsarilar et al.[1] Poly(ethylene glycol)$_{113}$ macromolecular chain transfer agent (PEG$_{113}$ macro-CTA) was prepared by adapting a literature protocol.[2] Methanolic silica sol (MA-ST) was kindly donated by Nissan Chemicals, Japan. The silica sol concentration was determined to be 34.7 w/w % using an Ohaus MB45 moisture analyzer. Deionized water was obtained from an in-house Elgastat Option 3A water purification unit. All solvents were obtained from Sigma-Aldrich (UK).

1.2. Synthesis of poly(methacrylic acid)$_{69}$ (PMAA$_{69}$) macro-CTA

Two types of chain transfer agents (CTAs) were used to prepare two PMAA macro-CTAs. 4-Cyano-4-(2-phenylethane sulfanylthiocarbonyl)sulfanylpentanoic acid (PETTC) chain transfer agent was used for the synthesis of a PMAA$_{69}$ macro-CTA. A round-bottomed flask was charged with MAA (5.00 g; 58 mmol), PETTC (0.28 g; 0.83 mmol), ACVA (46.5 mg, 0.166 mmol, [PETTC]/[ACVA] = 5.0) and ethanol (5.00 g). The sealed reaction vessel was purged with nitrogen and placed in a pre-heated oil bath at 70°C for 3 h. The resulting PMAA

macro-CTA was dialyzed first against a 9:1 water/methanol mixture and then against deionized water. The purified polymer was isolated by lyophilization. A mean DP of 69 was calculated for this macro-CTA using ^{1}H NMR spectroscopy by comparing the integrated signal intensity assigned to the aromatic protons at 7.2-7.4 ppm with that due to the methacrylic backbone at 0.4-2.5 ppm. After exhaustive methylation using excess trimethylsilyldiazomethane, THF GPC analysis of this $PMAA_{69}$ macro-CTA indicated an M_n of 9 700 g mol^{-1} and an M_w/M_n of 1.16.

1.3. Synthesis of poly(methacrylic acid)$_{62}$ ($PMAA_{62}$) macro-CTA

$PMAA_{62}$ macro-CTA was prepared using a commercially-available RAFT CTA (CPCP). A round-bottomed flask was charged with MAA (20.0 g; 232 mmol), CPCP (0.928 g; 3.32 mmol), ACVA (186.1 mg, 0.664 mmol, [CPCP]/[ACVA] = 5.0) and ethanol (31.4 g). The sealed reaction vessel was purged with nitrogen and placed in a pre-heated oil bath at 70°C for 3 h. The resulting PMAA macro-CTA was dialyzed first against a 9:1 water/methanol mixture and then against deionized water. The purified polymer was isolated by lyophilization. A mean DP of 62 was calculated for this macro-CTA using ^{1}H NMR spectroscopy by comparing the integrated signal intensity assigned to the aromatic protons at 7.3-8.0 ppm with that due to the methacrylic backbone at 0.4-2.5 ppm. After exhaustive methylation using excess trimethylsilyldiazomethane, THF GPC analysis indicated an M_n of 8 000 g mol^{-1} and an M_w/M_n of 1.16.

1.4. Synthesis of poly(glycerol monomethacrylate)$_{51}$ ($PGMA_{51}$) macro-CTA

CPDB RAFT agent (0.46 g, 2.0 mmol, ~ 80% purity), ACVA initiator (0.117 g, 0.40 mmol, [CPDB]/[ACVA] = 5.0) and anhydrous ethanol (30.0 g) were added to a round-bottomed

flask containing a magnetic stirrer bar. Once the CPDB and ACVA were fully dissolved, GMA monomer (20.0 g, 0.125 mol) was added to target a degree of polymerization (DP) of 60. This pink solution was sparged with N_2 for 30 min, before being placed into a pre-heated oil bath set at 70 °C. The GMA polymerization was quenched after 2 h by cooling the flask in ice, followed by exposure to air. The crude polymer was purified twice by precipitating into a ten-fold excess of dichloromethane. The precipitate was redissolved in water and the final macro-CTA was obtained in powdered form by lyophilization. ^{1}H NMR spectroscopy indicated a DP of 51 for this PGMA macro-CTA by comparing the integrated signals assigned to the five methacrylic backbone protons (0.4 to 2.5 ppm) with the integrated aromatic signals corresponding to the five protons associated with the RAFT CTA end-group. DMF GPC analysis (vs. poly(methyl methacrylate) standards) indicated M_n and M_w/M_n values of 12 900 g mol^{-1} and 1.18, respectively.

1.5. In situ encapsulation of silica sols within poly(methacrylic acid)$_{69}$-poly(benzyl methacrylate)$_{200}$ (PMAA$_{69}$-PBzMA$_{200}$) vesicles during polymerization-induced self-assembly (PISA)

$PMAA_{69}$ macro-CTA (0.10 g, 0.016 mmol), ACVA initiator (0.90 mg, 3.2 μmol, CTA/ACVA molar ratio = 5.0), ethanol (1.693 g) were weighed in turn into a reaction flask containing a magnetic stirrer bar. Then methanolic silica sol (0.953 g, silica concentration = 34.7 wt. %) and BzMA monomer (0.564 g, 3.18 mmol) was added to afford a 10 % w/w silica dispersion. The flask was sealed and degassed via N_2 for 30 min at 0 °C before being immersed in a 70 °C oil bath for 24 h. ^{1}H NMR spectroscopy indicated more than 99% BzMA conversion. The excess silica nanoparticles were removed by successive centrifugation-redispersion cycles (7 000 rpm, 15 min). For the first five cycles, each supernatant was replaced with a 73:23 ethanol/methanol mixture and for the final two cycles each supernatant was replaced with deionized water.

1.6. In situ encapsulation of fluorescein within poly(methacrylic acid)$_{62}$-poly(benzyl methacrylate)$_{300}$ (PMAA$_{62}$-PBzMA$_{300}$) vesicles during polymerization-induced self-assembly (PISA)

PMAA$_{62}$ macro-CTA (56.0 mg, 10 μmol), ACVA initiator (0.56 mg, 2.0 μmol, CTA/ACVA molar ratio = 5.0), fluorescein (1.66 mg, 5 μmol), ethanol (1.756 g) were successively weighed into a reaction flask containing a magnetic stirrer bar. Then BzMA monomer (0.529 g, 3 mmol) was added and the flask was sealed, cooled to 0 °C and degassed using N_2 for 30 min before being immersed in a 70 °C oil bath for 24 h. ^{1}H NMR spectroscopy indicated more than 99% BzMA conversion. The fluorescein encapsulation efficiency is ~ 36%, as determined by UV-vis spectroscopy. Excess fluorescein was removed by dialysis against water using dialysis tubing with a molecular weight cut-off of 3500 Da. Dialysis was judged to be complete when fluorescein could no longer be detected by visible absorption spectroscopy.

1.7. Synthesis of poly(ethylene glycol)$_{113}$-poly(2-hydroxypropyl methacrylate)$_{350}$ (PEG$_{113}$-PHPMA$_{300}$) vesicles via polymerization-induced self-assembly (PISA)

PEG$_{113}$ macro-CTA (100 mg, 18.7 μmol), VA-044 initiator (1.2 mg, 3.7 μmol, CTA/VA-044 molar ratio = 5.0) were dissolved in water (8.2 g) in a round-bottomed flask containing a magnetic stir bar. Afterwards, HPMA monomer (0.81 g, 5.6 mmol, target DP = 300) were added. The flask was sealed using a rubber septum and purged with nitrogen for 30 min and the RAFT aqueous dispersion polymerization of HPMA was conducted at 50 °C for 4 h.

1.8. Synthesis of poly(glycerol monomethacrylate)$_{51}$-poly(2-hydroxypropyl methacrylate)$_{350}$ (PGMA$_{51}$-PHPMA$_{350}$) vesicles via polymerization-induced self-assembly (PISA)

PGMA$_{51}$ macro-CTA (0.25 g, 29.4 μmol) and ACVA initiator (1.65 mg, 5.89 μmol, CTA/ACVA molar ratio = 5.0) were dissolved in 15.6 g water in a round-bottomed flask

containing a magnetic stir bar. Then HPMA monomer (1.485 g, 10.3 mmol, target DP = 350) was added and the flask was sealed using a rubber septum and purged with nitrogen for 30 min. The RAFT aqueous dispersion polymerization of HPMA was conducted at 70°C for 4 h.

1.9. Precipitation of calcite crystals in the presence of cargo-loaded vesicles

An aqueous solution (10 mL) comprising $CaCl_2$ (1.5 mM) and 0.10 % w/w vesicles was placed in a dessicator. $CaCO_3$ crystals were precipitated onto a glass slide placed at the base of this aqueous solution by exposure to ammonium carbonate vapor (2-3 g, placed at the bottom of the dessicator) for 24 h at 20 ºC. Then the glass slide was removed from the solution and washed three times with deionized water followed by three rinses with ethanol. Each occlusion experiment was repeated at least twice and consistent results were obtained in each case.

2. Characterization

2.1. ^{1}H NMR spectroscopy

^{1}H NMR spectra were recorded using a Bruker Avance 400 spectrometer operating at 400 MHz using either CD_3OD or d_8-THF solvents.

2.2. Gel permeation chromatography (GPC)

For THF GPC studies, the carboxylic acid groups on the PMAA macro-CTA were methylated using trimethylsilyldiazomethane, as reported by Couvreur et al.[3] The GPC set-up consisted of two 5 μM Mixed C columns connected to a WellChrom K-2301 refractive index detector. The mobile phase was HPLC-grade THF containing 1.0% glacial acetic acid and

0.05% w/v butylhydroxytoluene (BHT) at a flow rate of 1.0 mL min^{-1}. Molecular weights were calculated with respect to a series of near-monodisperse poly(methyl methacrylate) standards.

The DMF GPC set-up was operated at 60 °C with the instrument comprising two Polymer Laboratories PL gel 5 μm Mixed C columns and one PL polar gel 5 μm guard column connected in series to a Varian 390-LC multi-detector suite (refractive index detector only) and a Varian 290-LC pump injection module. The GPC eluent was HPLC-grade DMF containing 10 mM LiBr and was filtered prior to use. The flow rate was 1.0 mL min^{-1} and DMSO was used as a flow-rate marker. Calibration was conducted using a series of ten near-monodisperse poly(methyl methacrylate) standards ($M_n = 6.25 \times 10^2 – 6.18 \times 10^5$ g mol^{-1}, $K = 2.094 \times 10^{-3}$, $\alpha = 0.642$). Chromatograms were analyzed using Varian Cirrus GPC software.

2.3. Dynamic light scattering (DLS)

DLS measurements were conducted using a Malvern Zetasizer NanoZS instrument by detecting back-scattered light at an angle of 173°. Aqueous dispersions of the samples were diluted to 0.1 % w/w using deionized water. Hydrodynamic diameters were calculated using the Stokes-Einstein equation.

2.4. Optical microscopy

Optical microscopy images were recorded using a Motic DMBA300 digital biological microscope equipped with a built-in camera and analyzed using Motic Images Plus 2.0 ML software.

2.5. Transmission electron microscopy (TEM)

TEM images were obtained by depositing 0.15 % w/v alcoholic vesicle dispersions (or an aqueous vesicle dispersion in the presence of 6 mM $CaCl_2$) onto palladium-copper grids (Agar Scientific, UK) coated with carbon film. The grids were treated with a plasma glow discharge for approximately 30 seconds to create a hydrophilic surface prior to addition of a 5 μL droplet of the dispersion using a micropipet. Excess solvent was removed via blotting and the grid was stained with uranyl formate for 30 seconds. Excess stain was removed via blotting and the grid was carefully dried under vacuum. Imaging was performed using a FEI Tecnai G2 Spirit instrument.

2.6. Scanning electron microscopy (SEM)

Samples were fractured by placing a clean glass slide on top of the calcite-coated glass slide, pressing down lightly and twisting one slide relative to the other. The resulting randomly-fractured calcite crystals were examined by scanning electron microscopy using either an Inspect F instrument after sputter-coating with gold (15 mA, 1.5 min) or imaged directly using a Nova NanoSEM 450 instrument without any sputter-coating. A relatively low accelerating voltage (2-5 kV) was applied in order to prevent sample charging. To image the vesicles, a droplet of an aqueous dispersion of vesicles was dried onto a clean glass slide and then coated with gold prior to imaging. Sample milling was performed using a FEI Helio G4 CX dual beam-high resolution monochromated FEG SEM instrument equipped with a focused ion beam (FIB). The operating voltage was 30 kV and the beam current was varied between 0.1 and 5 nA.

2.7. Small-angle X-ray scattering (SAXS)

SAXS patterns were collected at an international synchrotron source (ESRF, station ID02, Grenoble, France) using monochromatic X-ray radiation (λ = 0.0995 nm, with q ranging from 0.013 to 1.8 nm^{-1}, where q is the length of the scattering vector, i.e. $q = 4\pi \sin \theta/\lambda$ and θ is one-half of the scattering angle) and a Ravonix MX-170HS CCD detector. Glass capillaries of 2.0 mm diameter were used as sample holders. Scattering data were reduced using standard software packages available at the beamline and were further analyzed using Irena SAS macros for Igor Pro.[4] Water was used for the absolute intensity calibration.

2.8. Other measurements

Fluorescence microscopy images were recorded on a Zeiss Axio Scope A1 microscope fitted with an AxioCam 1Cm1 monochrome camera. Images were captured and processed using ZEN lite 2012 software. Confocal fluorescence images were recorded using a Nikon A1 microscope equipped with Nikon elements software. Raman spectra were recorded using a Renishaw 2000 Raman microscope equipped with a 785 nm diode laser. Thermogravimetric analysis (TGA) was conducted from 20 °C to 900 °C in air using a Perkin-Elmer Pyris 1 instrument at 10 °C min^{-1}.

Table S1. Summary of the diameter and zeta potential of the silica nanoparticles and various vesicles.

Sample	D_{DLS}[a] (nm)	D_{EM}[b] (nm)	Zeta potential (mV)
Silica nanoparticles	50 ± 43	12 ± 2.7[c]	-45 ± 7
$PMAA_{69}$-$PBzMA_{200}$ vesicles	191 ± 76	181 ± 53	-53 ± 5
Silica-loaded $PMAA_{69}$-$PBzMA_{200}$ vesicles	232 ± 60	208 ± 46	-50 ± 6
Fluorescein-loaded $PMAA_{62}$-$PBzMA_{300}$ vesicles	647 ± 193	637 ± 178	-48 ± 7

[a] Diameter determined by DLS; [b] Diameter determined by TEM or SEM; [c] Diameter determined by TEM.

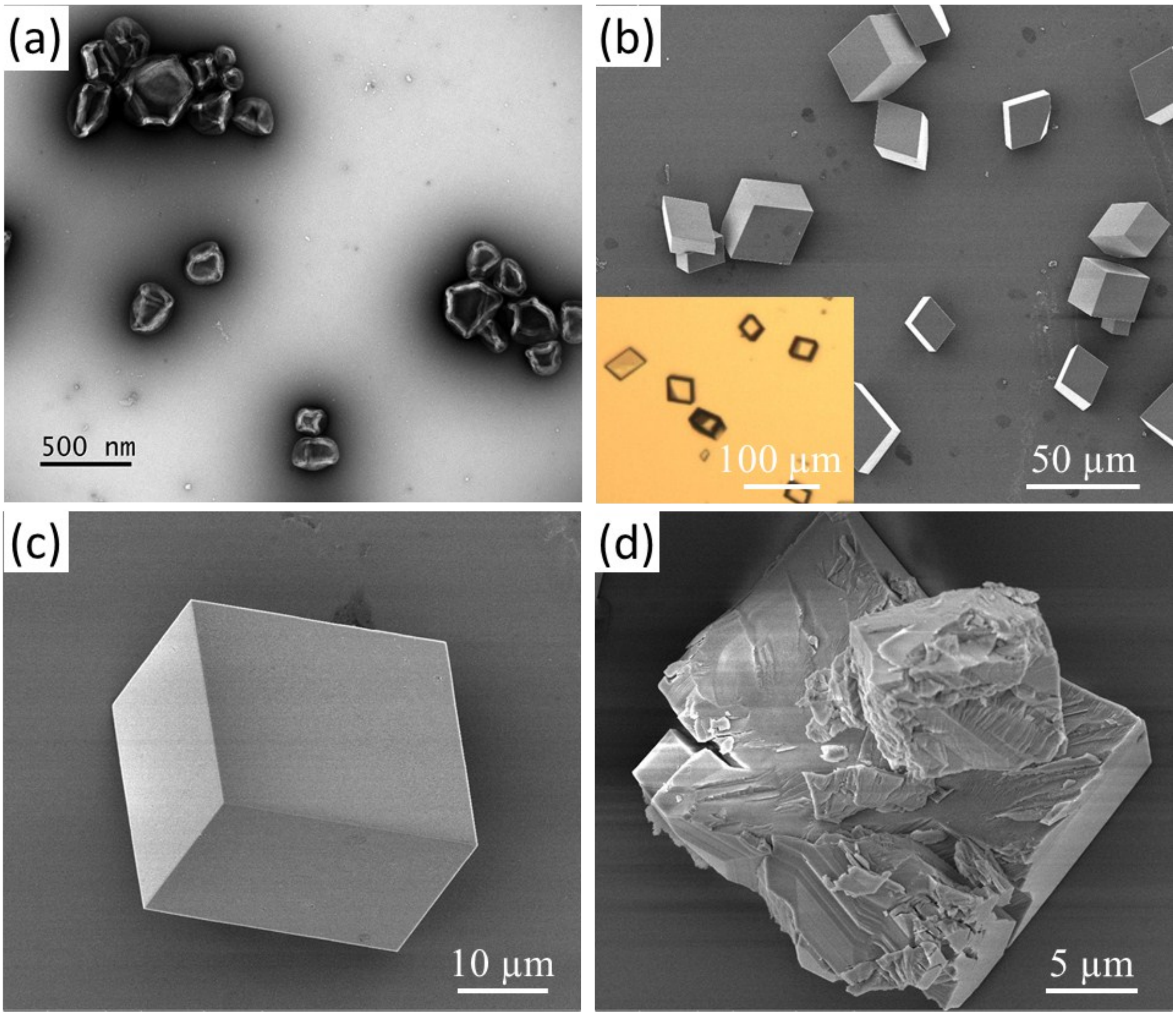

Fig. S1. Calcite crystals precipitated in the presence of 0.1 % w/w PEG_{113}-$PHPMA_{300}$ vesicles. (a) TEM image recorded for the PEG_{113}-$PHPMA_{300}$ vesicles alone. (b) SEM image recorded for calcite crystals precipitated in the presence of PEG_{113}-$PHPMA_{300}$ vesicles. Inset shows the corresponding optical microscopy image. (c) SEM image of the same rhombahedral calcite crystals recorded at higher magnification. (d) SEM image showing the internal structure of a randomly-fractured calcite crystal, indicating no evidence for any vesicle occlusion in this control experiment.

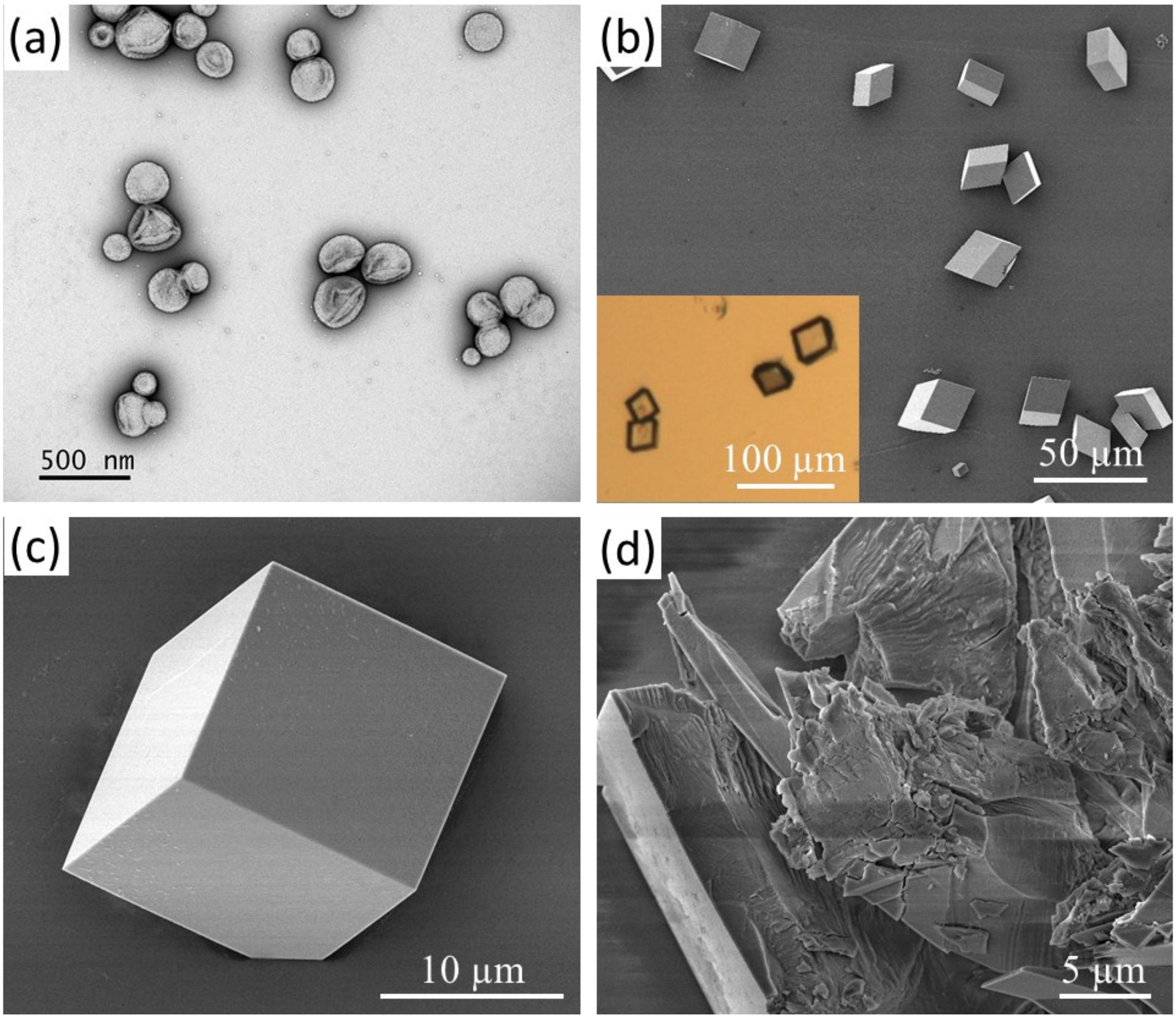

Fig. S2. Calcite crystals precipitated in the presence of 0.10 % w/w $PGMA_{51}$-$PHPMA_{350}$ vesicles. (a) TEM image recorded for $PGMA_{51}$-$PHPMA_{350}$ vesicles alone; (b) SEM image recorded for calcite crystals precipitated in the presence of $PGMA_{51}$-$PHPMA_{350}$ vesicles. Inset shows the corresponding optical microscopy image. (c) SEM image of the same rhombahedral calcite crystals recorded at higher magnification. (d) SEM image showing the internal structure of a randomly-fractured calcite crystal, indicating no evidence for any vesicle occlusion in this control experiment.

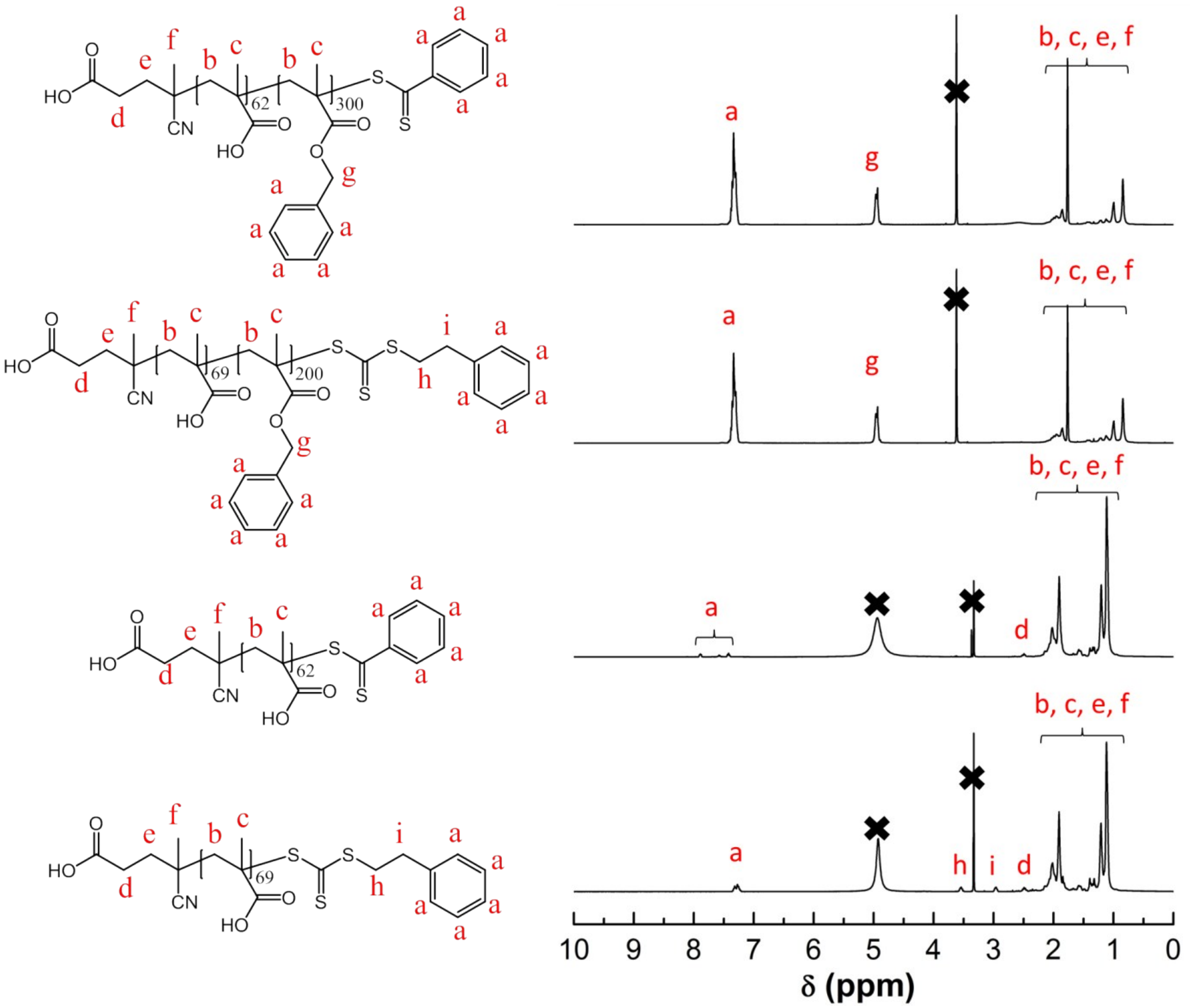

Fig. S3. Assigned ^{1}H NMR spectra recorded for two $PMAA_x$ macro-CTAs (in CD_3OD) and two $PMAA_x$-$PBzMA_y$ diblock copolymers (in d_8-THF).

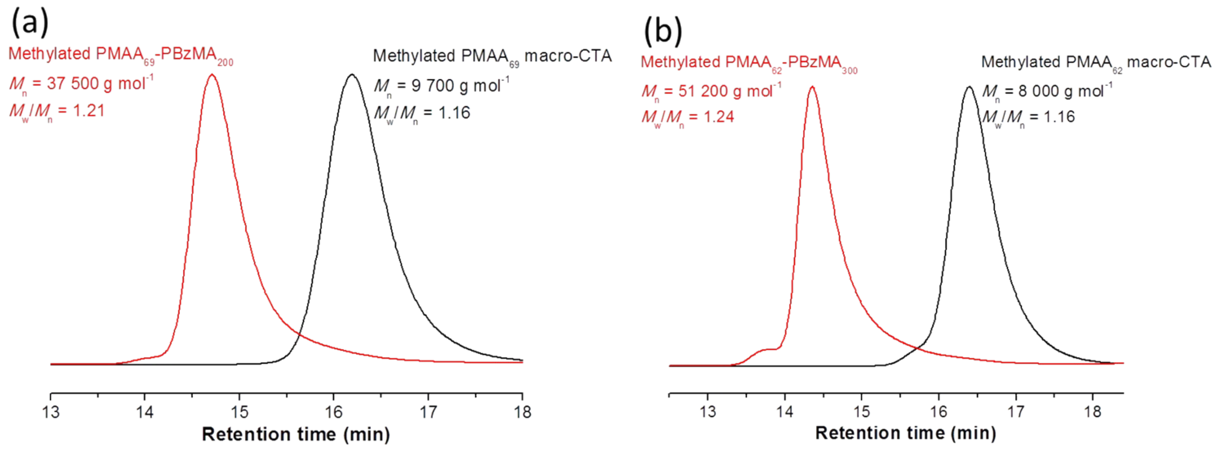

Fig. S4. THF GPC curves (vs. poly(methyl methacrylate) standards) recorded for methylated $PMAA_x$ macro-CTAs and their corresponding methylated $PMAA_x$-$PBzMA_y$ diblock copolymers. The carboxylic acid groups on the PMAA block were fully methylated using excess trimethylsilyldiazomethane. This methylation protocol led to some coupling between the copolymer chains and a weak high molecular weight shoulder is observed as a result of this side reaction. However, this has relatively little effect on the final M_w/M_n value.

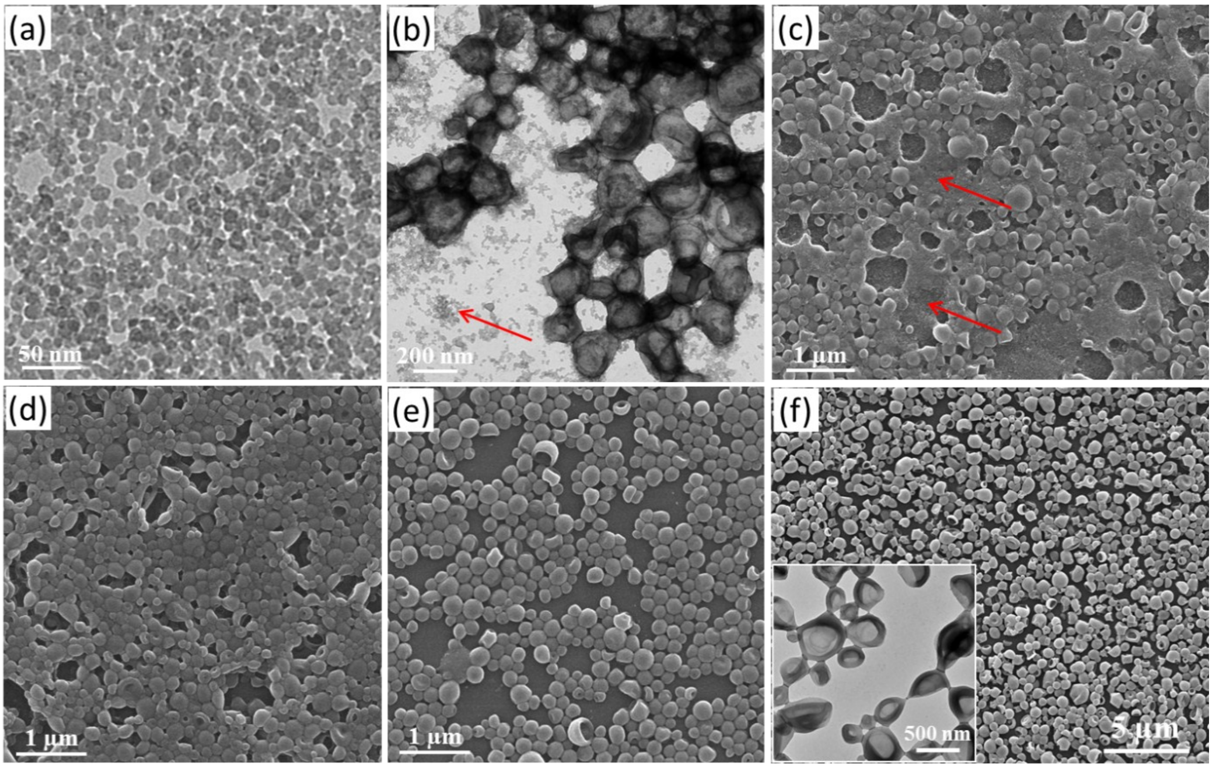

Fig. S5. TEM images recorded for (a) silica nanoparticles alone and (b) $PMAA_{69}$-$PBzMA_{200}$ vesicles prepared in the presence of the same silica nanoparticles prior to their purification via multiple centrifugation-redispersion cycles to remove excess non-encapsulated silica. (c) SEM image of $PMAA_{69}$-$PBzMA_{200}$ vesicles prepared in the presence of silica nanoparticles prior to purification; (d) SEM image of $PMAA_{69}$-$PBzMA_{200}$ vesicles prepared in the absence of silica nanoparticles; (e) SEM image of $PMAA_{69}$-$PBzMA_{200}$ vesicles prepared in the presence of silica nanoparticles after purification; (f) SEM image of $PMAA_{62}$-$PBzMA_{300}$ vesicles prepared in the presence of fluorescein after purification to remove excess non-encapsulated dye (the inset is a corresponding TEM image for the same sample). N.B. Red arrows shown in images (b) and (c) indicate excess non-encapsulated silica.

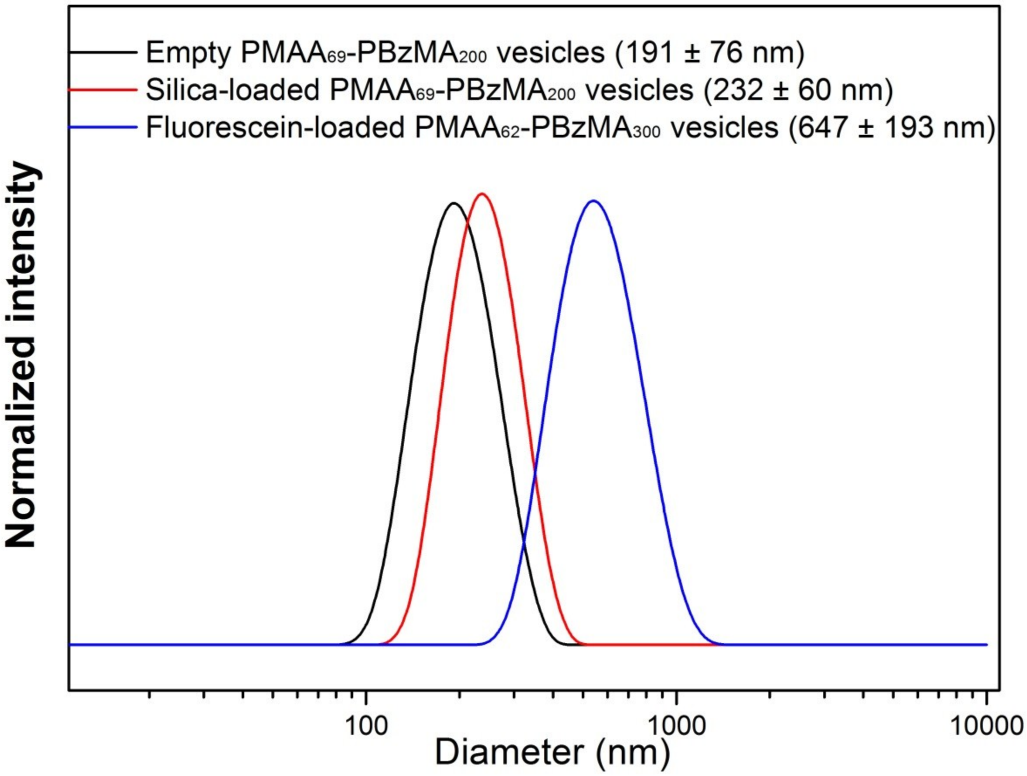

Fig. S6. DLS particle size distributions recorded for empty $PMAA_{69}$-$PBzMA_{200}$ vesicles, silica-loaded $PMAA_{69}$-$PBzMA_{200}$ vesicles and fluorescein-loaded $PMAA_{62}$-$PBzMA_{300}$ vesicles.

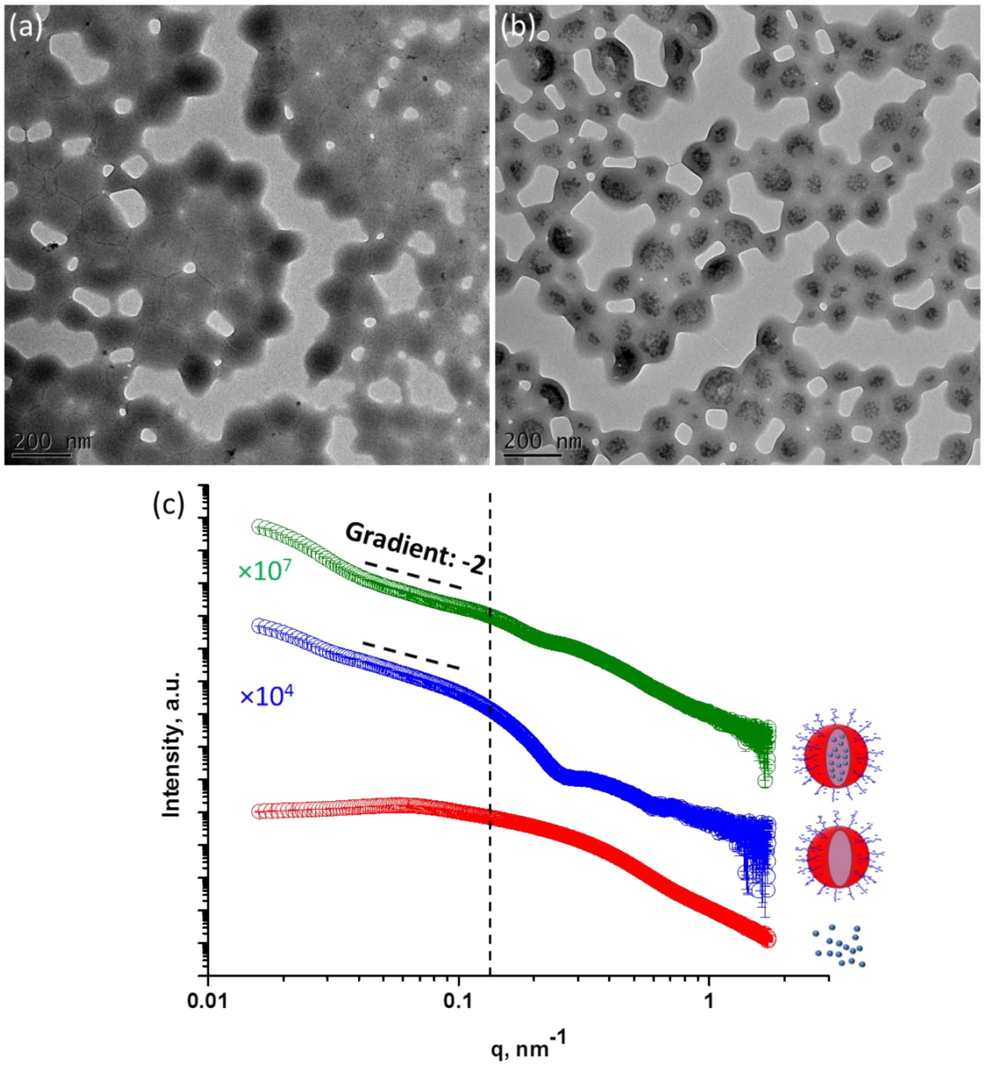

Fig. S7. TEM images recorded for (a) empty PMAA$_{69}$-PBzMA$_{200}$ vesicles and (b) silica-loaded PMAA$_{69}$-PBzMA$_{200}$ vesicles. [N.B. Neither TEM grid was stained prior to imaging]. (c) SAXS patterns obtained for silica nanoparticles (red circles), empty PMAA$_{69}$-PBzMA$_{200}$ diblock copolymer vesicles (blue circles) and silica-loaded PMAA$_{69}$-PBzMA$_{200}$ diblock copolymer vesicles (green circles). A methanolic silica sol was diluted to 1.0% w/w using ethanol to afford a 73:27 w/w ethanol/methanol mixture for SAXS measurements. The empty and silica-loaded PMAA$_{69}$-PBzMA$_{200}$ diblock copolymer vesicles were prepared via PISA in the presence of 0% or 10% w/w silica, respectively, before being diluted to 1.0% w/w in a 73:27 w/w ethanol/methanol mixture for SAXS measurements. For clarity, the upper two SAXS patterns are shifted vertically by arbitrary scaling factors (see labels). The corresponding schematic cartoons represent silica nanoparticles alone (bottom), empty PMAA$_{69}$-PBzMA$_{200}$ vesicles (middle) and silica-loaded PMAA$_{69}$-PBzMA$_{200}$ (top) vesicles. The vesicles shown in **Fig. S7a** and **S7b** were not stained, so their vesicular morphology is not readily discernible. However, uranyl formate staining of the same samples clearly revealed a well-defined vesicular morphology in each case (see **Fig. 1**). Indeed, this morphology was confirmed by the gradient of -2 observed in the low *q* range of each SAXS pattern shown in **Fig. S7c**.[5]

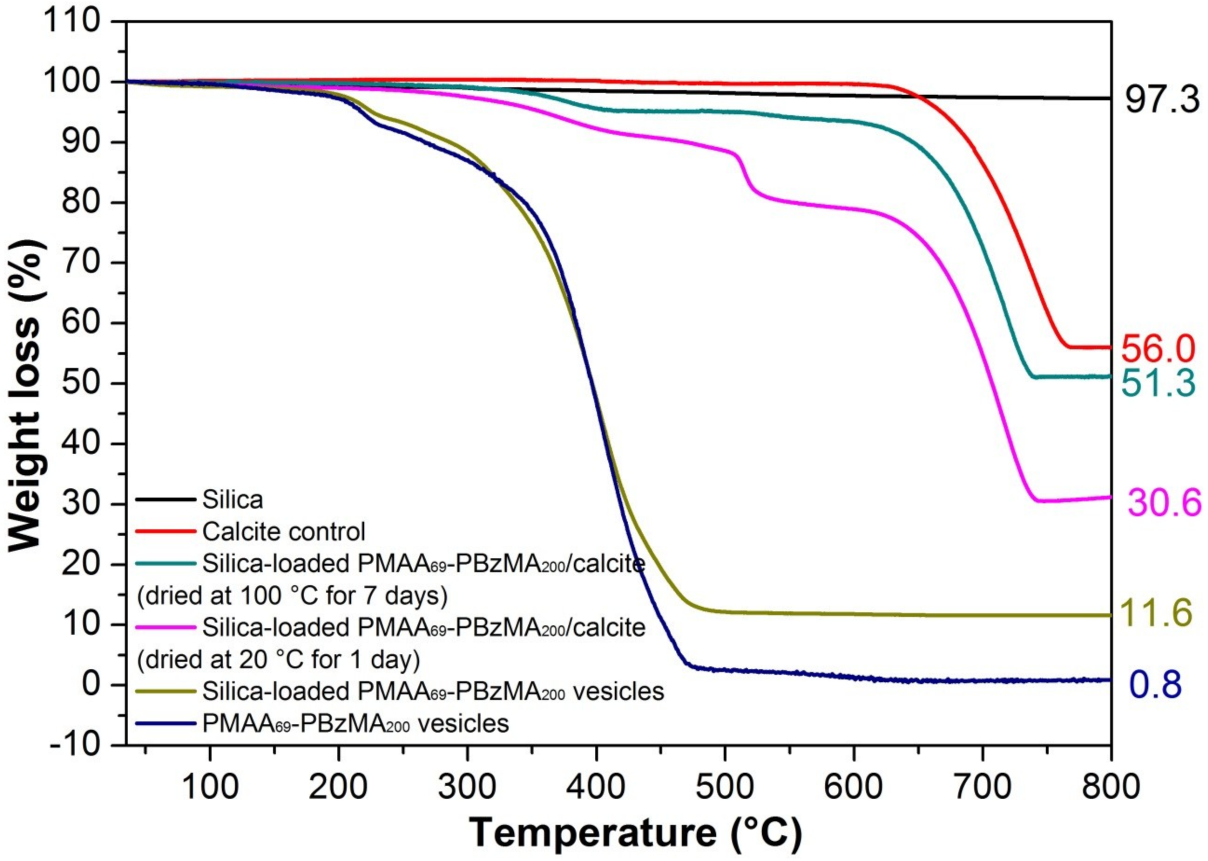

Fig. S8. Thermogravimetric analysis (TGA) curves recorded for the dried silica sol, a calcite control, silica-loaded $PMAA_{69}$-$PBzMA_{200}$/calcite nanocomposites, silica-loaded $PMAA_{69}$-$PBzMA_{200}$ vesicles and the empty $PMAA_{69}$-$PBzMA_{200}$ vesicles.

Calculation of the extent of occlusion of $PMAA_{69}$-$PBzMA_{200}$ vesicles within calcite

Based on TGA data (see **Fig. S8**), silica sols exhibited a relatively small weight loss (2.7 %) and only 0.8% ash was observed when heating $PMAA_{69}$-$PBzMA_{200}$ vesicles up to 800 °C in air. The onset of calcite decomposition occurs at 600 °C, giving a weight loss of 44 % (CO_2) and a residue of 56 % (CaO). This is in good agreement with the expected thermal decomposition reaction: $CaCO_3$ (s) → CaO (s) + CO_2 (g)↑. The aqueous lumen of the $PMAA_{69}$-$PBzMA_{200}$ vesicles remained intact when silica-loaded $PMAA_{69}$-$PBzMA_{200}$/calcite nanocomposites were dried at 20°C for 24 h (see the corresponding TGA curve shown in **Fig. S8**). Unfortunately, uncertainty in the amount of water remaining within the vesicle lumen prevents calculation of the extent of occlusion of silica-loaded $PMAA_{69}$-$PBzMA_{200}$ within

calcite from the above data. However, silica-loaded $PMAA_{69}$-$PBzMA_{200}$/calcite nanocomposites can be completely dried on heating to 100 °C for seven days. This relatively mild heat treatment removes all the encapsulated water within the vesicles, so the resulting nanocomposites comprise solely silica, $PMAA_{69}$-$PBzMA_{200}$ vesicles and calcite ($CaCO_3$).

Calculation of the silica content of the vesicles in mass % from the TGA data shown in **Fig. S8** requires various corrections. The dried methanolic silica sol exhibited a small mass loss (2.7 %) on heating and the vesicles alone exhibited a small amount of ash (0.8%) after pyrolysis at ~600 °C. Thus the total ash in mass %, A, obtained after pyrolysis of the silica-loaded $PMAA_{69}$-$PBzMA_{200}$ vesicles, is given by the following equation:

$$A = \frac{Silica\ \times\ 97.3\%\ + Vesicle\ \times\ 0.8\%}{Silica\ + Vesicle}$$

Inspecting the TGA curve obtained for silica-loaded vesicles shown in **Fig. S8**, A = 11.6%. Hence the silica mass % within these vesicles can be calculated as follows:

$$Silica\ content\ of\ the\ vesicles\ in\ mass\% = \frac{Silica}{Silica + Vesicle} = 11.2\ \%$$

Assuming that the relative mass contents % for the $PMAA_{69}$-$PBzMA_{200}$ vesicles, silica, CaO and CO_2 are x, y, m, and n, respectively, then the following four equations can be obtained:

$0.8\% \times x + 97.3\% \times y + m = 51.3$ (1)

$99.2\% \times x + \mathrm{n} = 48.7$ (2)

$56 \times n = 44 \times m$ (3)

$0.8\% \times x + 97.3\% \times y = 11.6\% \times (x + y)$ (4)

Solving the above equations, $x = 9.4$, $y = 1.2$ and $m = 50.1$, $n = 39.3$

So, the extent of vesicle occlusion within the calcite is 9.4% by mass.

The cartoon below shows the outer radius (R) and inner radius (r) of the vesicles. The mean vesicle diameter (2R) can be estimated by analyzing SEM images and the vesicle membrane thickness was calculated by TEM analysis. For the silica-loaded $PMAA_{69}$-$PBzMA_{200}$ vesicles, the vesicle diameter = 208 ± 46 nm and the membrane thickness (*T*) = 19 ± 3 nm.

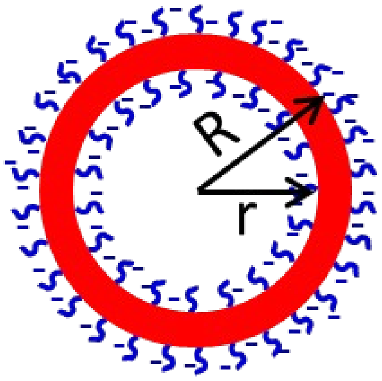

Then the effective density of empty vesicles, $\rho_{effective}$, can be calculated as follows.

$$\rho_{effective} = \frac{(R^3 - r^3) \times \rho_{vesicle}}{R^3}$$

The solid-state density of the dried $PMAA_{69}$-$PBzMA_{200}$ vesicles $(\rho_{vesicle})$ was determined to be 1.3 g cm^{-3} at 20 °C by helium pycnometry (Micrometrics AccuPyc 1330 helium pycnometer). Given the extent of occlusion by *mass* (x), the extent of vesicle occlusion by *volume* $(x^{'})$ can be calculated using the following equation:

$$x^{'} = \frac{\frac{xR^3}{(R^3 - r^3)\rho_{vesicle}}}{[\frac{xR^3}{(R^3 - r^3)\rho_{vesicle}} + \frac{m + n}{\rho_{calcite}}]} \times 100\ \%$$

Thus the extent of vesicle occlusion was calculated to be 32.6% (or approximately 33%) by volume.

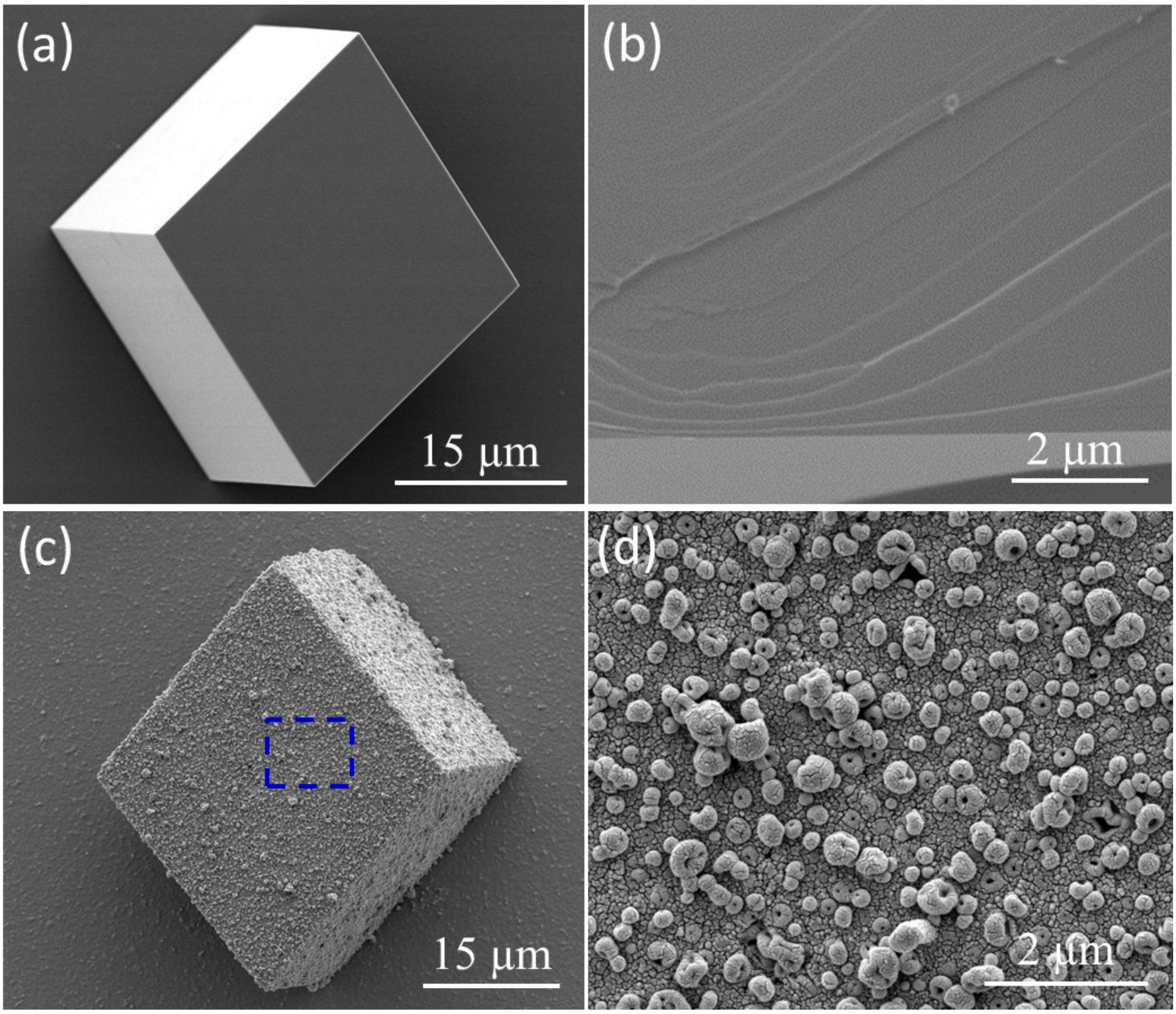

Fig. S9. (a) Calcite crystals precipitated in the absence of any vesicles; (b) same crystals after their random fracture, which confirms their featureless internal structure; (c) calcite crystals precipitated in the presence of 0.1 % w/w silica-loaded $PMAA_{69}$-$PBzMA_{200}$ vesicles; (d) high magnification SEM image showing the surface morphology of the calcite crystal shown in (c). Note the dense surface layer of embedded vesicles.

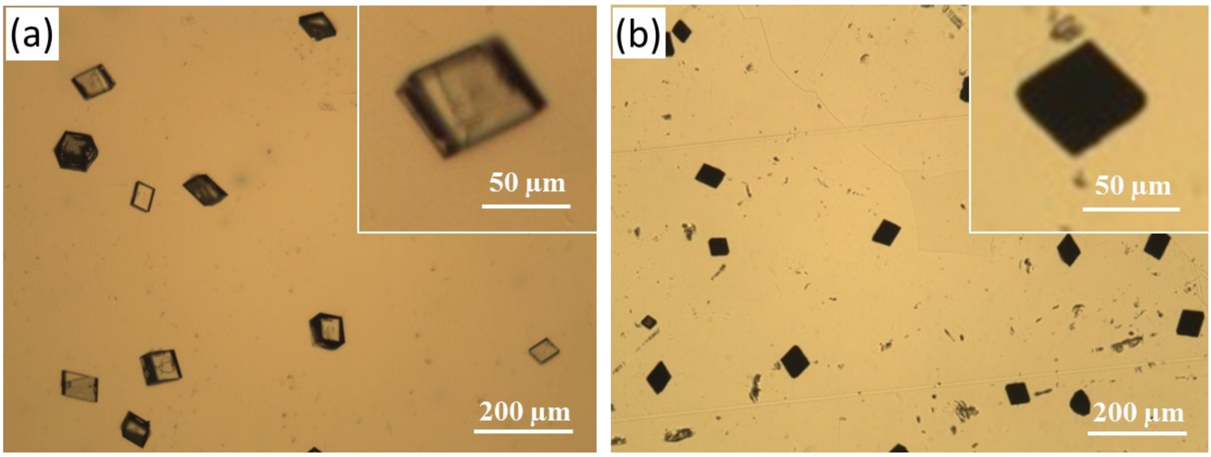

Fig. S10. Optical micrographs recorded for (a) calcite precipitated in the absence of any additives; (b) calcite precipitated in the presence of silica-loaded vesicles [Ca^{2+} concentration: 1.5 mM, 0.10% w/w silica-loaded vesicles]. The insets each show the corresponding higher magnification images.

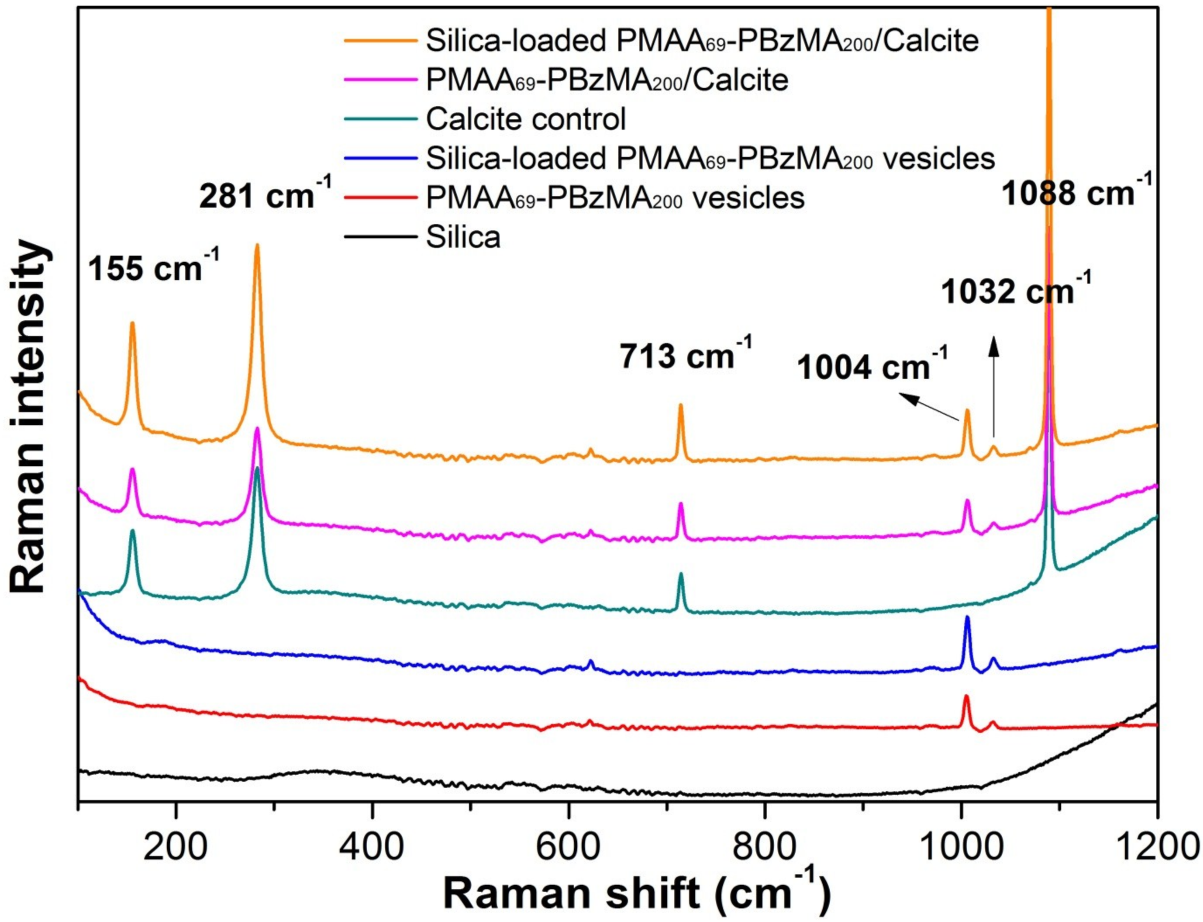

Fig. S11. Raman spectra recorded for the following samples (from bottom to top): silica sol (black spectrum), dried $PMAA_{69}$-$PBzMA_{200}$ vesicles alone (red spectrum), dried silica-loaded $PMAA_{69}$-$PBzMA_{200}$ vesicles (blue spectrum), calcite alone (green spectrum), $PMAA_{69}$-$PBzMA_{200}$/calcite (pink spectrum), silica-loaded $PMAA_{69}$-$PBzMA_{200}$/calcite (orange spectrum).

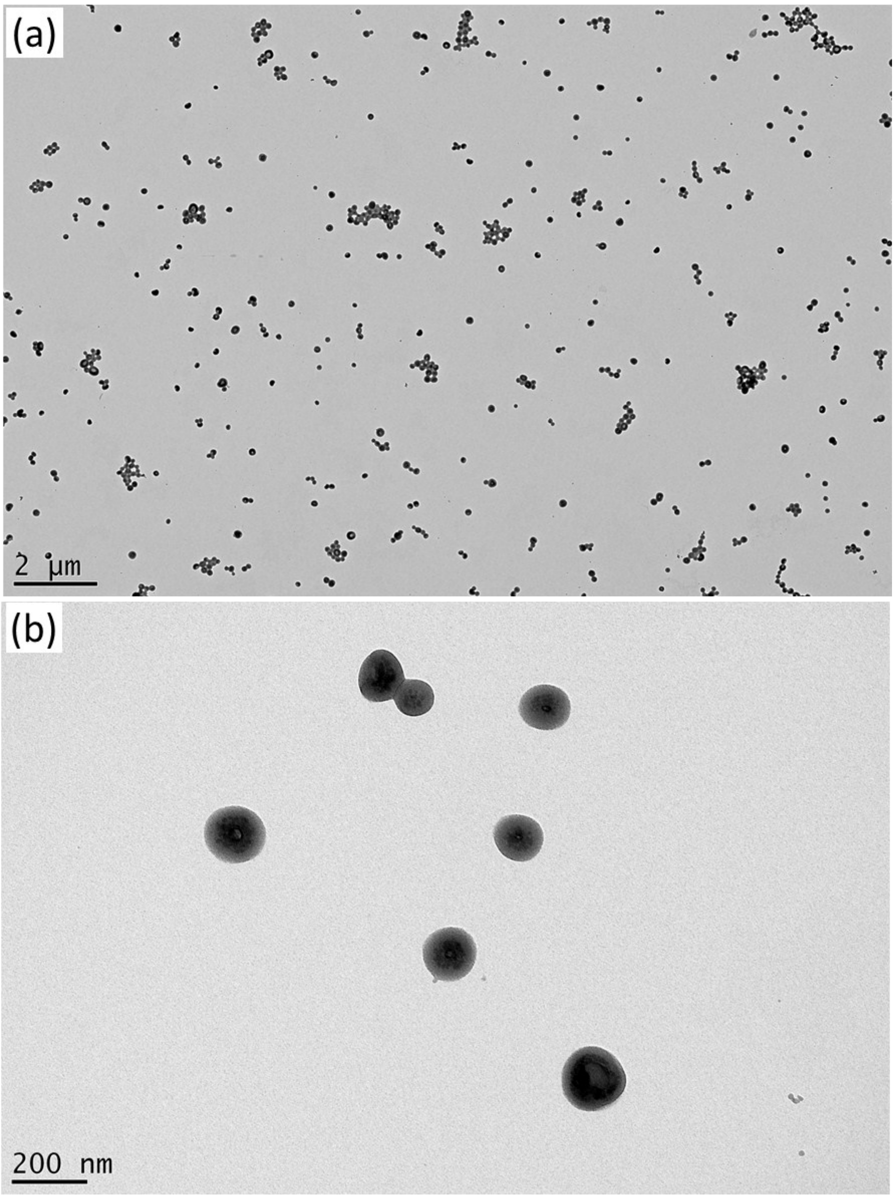

Fig. S12. TEM images recorded for silica-loaded $PMAA_{69}$-$PBzMA_{200}$ vesicles. This aqueous dispersion was diluted using a 6 mM $CaCl_2$ aqueous solution prior to TEM grid preparation to minimize incipient flocculation of the vesicles.

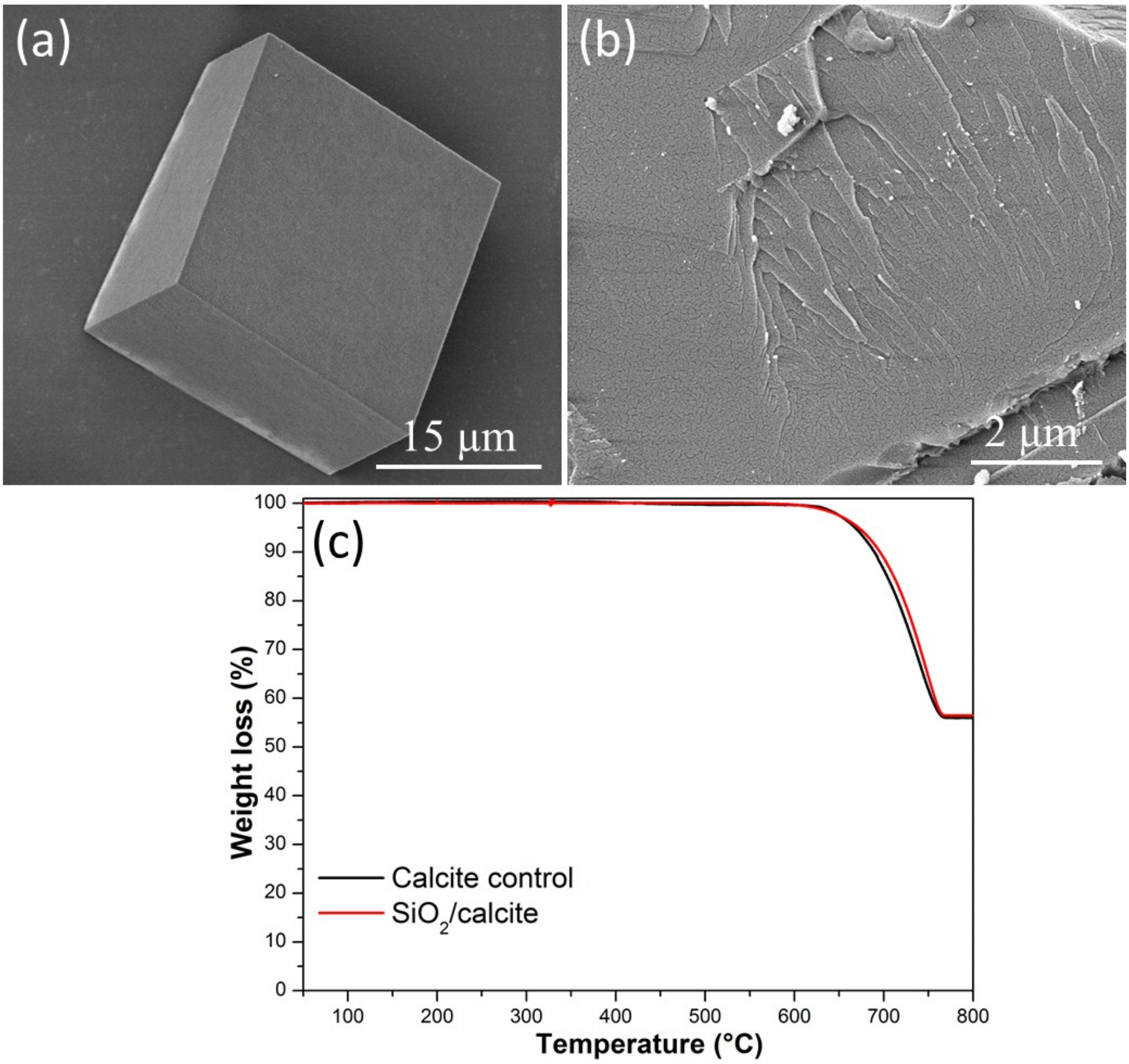

Fig. S13. (a) Calcite crystals precipitated in the presence of 0.10% w/w silica nanoparticles; (b) magnified SEM image showing internal cross-section; (c) TGA curves recorded for calcite control and calcite crystals precipitated in the presence of silica nanoparticles alone (no $PMAA_{69}$-$PBzMA_{200}$ vesicles). Both SEM and TGA analyses suggest that the occlusion of silica nanoparticles alone within calcite is negligible in the absence of any $PMAA_{69}$-$PBzMA_{200}$ vesicles.

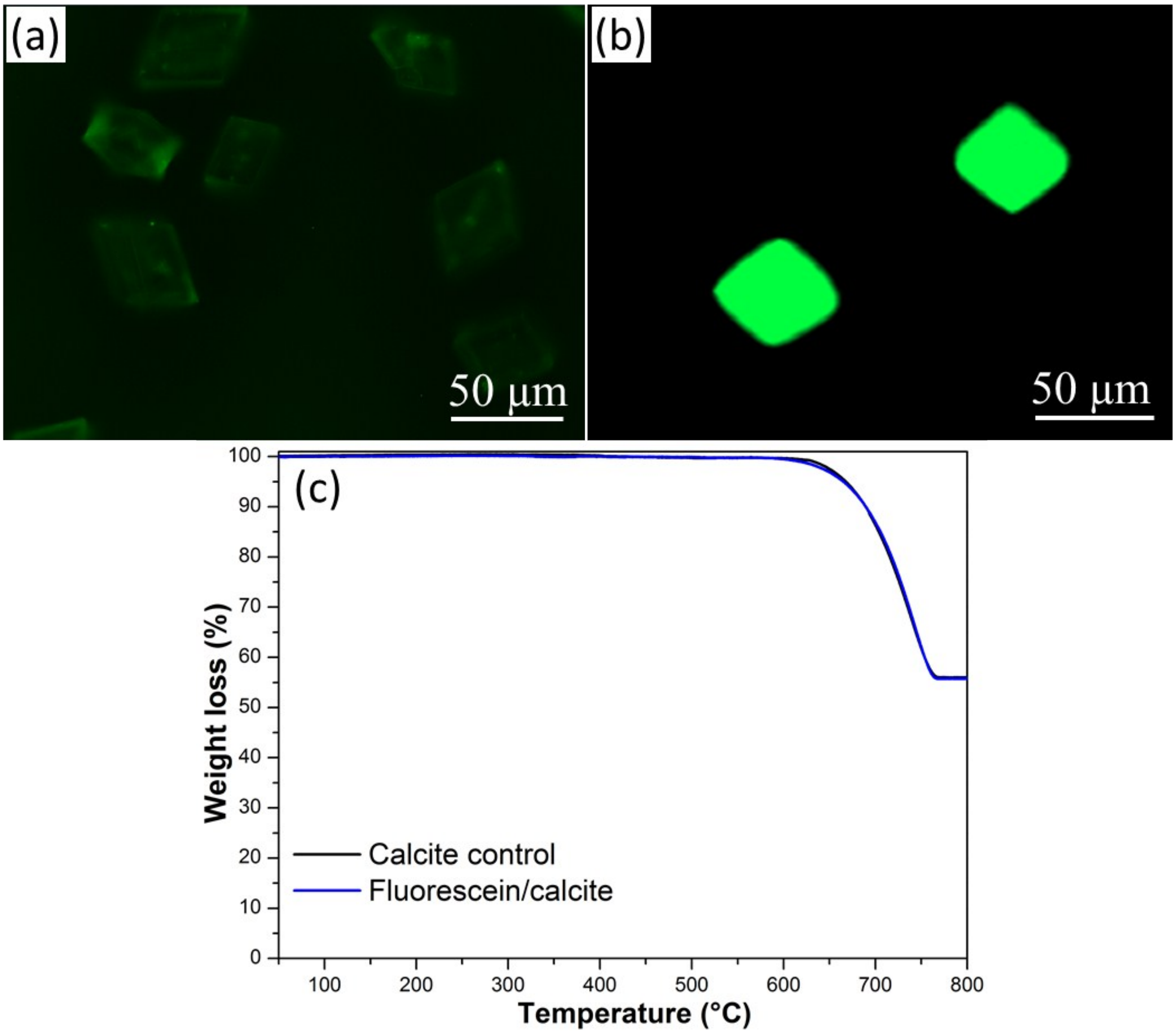

Fig. S14. (a) Fluorescence microscopy images recorded for calcite crystals precipitated in the presence of 0.001% w/w fluorescein (N.B. It is not possible to prepare a 0.1% w/w aqueous soluion of fluorescein owing to the relatively low water solubility of this dye. Nevertheless, the fluorescein concentration of 0.001% w/w is significantly higher than that within the fluorescein-loaded vesicles). (b) Fluorescence microscopy images recorded for calcite crystals precipitated in the presence of 0.1% w/w fluorescein-loaded $PMAA_{62}$-$PBzMA_{300}$ vesicles. (c) TGA curves recorded for a pure calcite control and also for calcite prepared in the presence of 0.001% w/w fluorescein.

Based on **Fig. S14a**, the fluorescein appears to merely adsorb onto the surface of the calcite crystals. It is not possible to tell whether any fluorescein is incorporated within the crystals from such images. However, TGA analysis suggests that the the extent of fluorescein occlusion must be negligible. In contrast, for calcite prepared in the presence of 0.1% w/w fluorescein-loaded vesicles, the whole crystal is highly fluorescent, indicating the uniform

occlusion of such vesicles within the calcite crystals (see **Fig. S14b**). This is confirmed by SEM analysis and confocal microscopy studies, see **Fig. 3**.

References

1 M. Semsarilar, E. R. Jones, A. Blanazs and S. P. Armes, *Adv. Mater.*, 2012, **24**, 3378.

2 N. J. Warren, O. O. Mykhaylyk, D. Mahmood, A. J. Ryan and S. P. Armes, *J. Am. Chem. Soc.*, 2014, **136**, 1023.

3 L. Couvreur, C. Lefay, J. Belleney, B. Charleux, O. Guerret and S. Magnet, *Macromolecules,* 2003, **36**, 8260.

4 J. Ilavsky, P. R. Jemian, *J. Appl. Crystallogr.,* 2009, **42**, 347.

5 M. J. Derry, L. A. Fielding, N. J. Warren, C. J. Mable, A. J. Smith, O. O. Mykhaylyk and S. P. Armes, *Chem. Sci.*, 2016, **7**, 5078.
